# Hospital discharge of the elderly-an observational case study of functions, variability and performance-shaping factors

**DOI:** 10.1186/1472-6963-14-365

**Published:** 2014-08-30

**Authors:** Kristin Laugaland, Karina Aase, Justin Waring

**Affiliations:** Health Trust Førde, Førde, Norway; Department of Health Studies, University of Stavanger, Stavanger, Norway; Regional Centre for Age-Related Medicine, Stavanger University Hospital, Stavanger, Norway; Nottingham University Business School, Nottingham, United Kingdom

**Keywords:** Hospital discharge, Elderly, Functions, Variability, Performance-shaping factors

## Abstract

**Background:**

Understanding and improving hospital discharge has assumed major importance since it represents an error-prone transition in care. One barrier to improvement is the lack of detailed understanding of how hospital discharge is organized, including its interdependencies and influential performance-shaping factors (PSFs). This study examines the discharge of elderly patients using the Functional Resonance Analysis Method, developed to analyze performance variability in complex systems. Our main aim was to identify hospital discharge functions, variability, and PSFs that may explain the variability and different outcomes in discharge practices by incorporating multiple-stakeholder perceptions (health-care providers, patients, next of kin).

**Methods:**

The data consisted of moderate participant observations of 20 elderly patients (>75) discharged from hospital to primary health care. The data comprised 90 hours’ observations at hospital wards, including 173 conversations with patients, next of kin, and health-care personnel involved in discharge.

**Results:**

We identified 10 common functions in the discharge of elderly patients to primary health care. We found substantial variability in terms of *timing*, *duration*, and *precision*. Duration is a significant source of variability, primarily determined by the time of day the patient was determined medically fit for discharge. Precision issues related to (1) decision-making criteria concerning the medical fitness decision and appropriate level of care, (2) quality of discharge planning, (3) degree of patient involvement, and (4) quality of information transfer. PSFs were temporal conditions (degree of time pressure), individual and team characteristics, patient factors, organizational factors (unit, specialization, leadership, institutionalized routines), work environment factors (bed availability, availability in municipal services, quality of discharge planning, familiarity with the patient, pressure from next of kin, doctor’s specialization) and regulatory influences (financial incentives).

**Conclusions:**

The study provides a detailed understanding of the discharge of elderly patients by describing common functions and variability in performance caused by multiple PSFs. Our findings indicate the necessity for studying multiple factors related to discharge, interdependencies, and their effects on a range of discharge outcomes incorporating a multiple-stakeholder perspective. We argue that the existing sequential approaches to the complexity surrounding hospital discharge are inadequate. Given the interdependencies among functions, there is a need for corresponding multi-factorial interventions.

**Electronic supplementary material:**

The online version of this article (doi:10.1186/1472-6963-14-365) contains supplementary material, which is available to authorized users.

## Background

Understanding and improving the process of hospital discharge has assumed major importance [1] since it represents an error-prone transition in care [2]. Elderly patients are notably at risk for adverse events in general and with transitions across health-care providers in particular [3–5]. In this regard, ineffective care processes, poor communication, and deficient documentation have been identified as major contributing factors [1, 6]. Despite efforts to improve hospital discharge, current evidence is scant and inconclusive, and progress toward improvement has been limited and slow [7].

Mainstream patient safety research has tended to be reactive: it investigates adverse events to identify cause-and-effect relationships, from which improvements can be formulated [8]. We argue that the existing, sequential approaches to the complexity of hospital discharge are inadequate. A barrier to improvement is the lack of detailed understanding as to how the process of hospital discharge is organized, including its interdependencies and contextual factors [7, 9, 10]. Little consideration has thus far been afforded to the inherent variability in everyday practice and how this can prospectively create system vulnerabilities [11]. Knowledge about performance variability has not commonly been recognized as an asset, and it has rarely been gathered in a systematic fashion [12].

In the health-care context, the wide variety in patients, their relatives, geographic settings, professional groups, and working conditions means that continuous adaptations are essential toward ensuring overall performance [13]. Variability thus represents a normal, necessary part of clinical work, and it demands the ability to cope with unpredictable, unstable working environments [14]. However, performance variability and the factors that influence hospital discharge practices and outcomes are for the most part poorly understood and have not been fully investigated.

This paper applies an integrated approach to the study of hospital discharge, focusing on functions, interdependencies, and performance-shaping factors from a multiple-stakeholder perspective. Qualitative observational case studies of the hospital discharge of elderly patients are used to identify functions and demonstrate the performance variability that surrounds hospital discharge practices by applying the Functional Resonance Analysis Method (FRAM). The FRAM is an innovative method that is developed to analyze performance variability in complex systems [14]. Specifically, the main aims of the paper are to identify;

- The functions of hospital discharge;
- The areas of variations within those functions, and;
- The performance shaping factors (PSFs) that may explain those variations.

To accomplish these aims we gather and incorporate the perceptions of not just healthcare providers, but patients and their next of kin.

Before introducing the case study and findings, we describe the characteristics and practical approach of the FRAM. We explain how it was used analytically to determine the details of elderly patient discharges in a Norwegian setting.

### Functional resonance analysis method

The FRAM is a systemic, non-linear approach that defines complex systems in terms of both their overall and constituent functions. “Functions” here refers to the activities or sets of activities that are necessary to produce a particular outcome, e.g., hospital discharge of the elderly. The aim is to identify and assess the interdependencies among functions within complex systems. In practice, this involves a description of what individuals or groups do to achieve their functional aim—as opposed to analyzing prescribed models of behaviors, e.g., standard operating procedures or care pathways [14]. The FRAM clarifies outcomes in terms of how functions become connected, how everyday performance variability may result from the way individual functions are completed, and how these functions affect one another. In this regard, a system is a set of coupled or mutually dependent functions [14].

**Figure 1:**
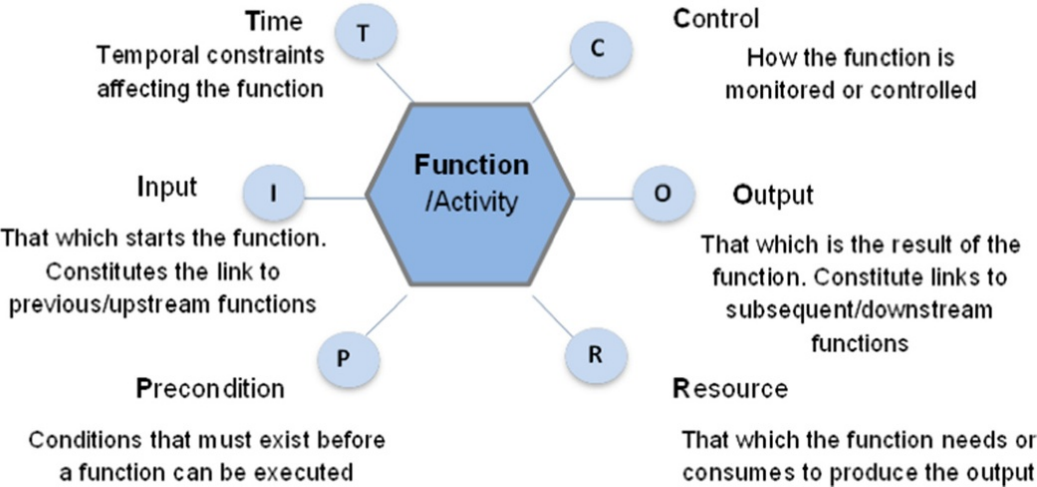
**Describing a function by six aspects** **[** [11] **]** **:p.46.**

In practical terms, the FRAM consists of a five-step approach [14]. The first step involves deciding the purpose of the FRAM analysis, i.e., the clinical work under examination. The second step is identifying the functions that are necessary for that work to be accomplished (as defined by the participants involved in the activity) as well as describing each function in terms of six basic aspects (output, input, precondition, resource, control, and time), as illustrated in Figure 1. The third step involves identification and description of variability in the identified functions in addition to a consideration of the manner and reason for their variation. The fourth step is that of determining how variability within one function affects other functions and how such effects spread across the system in the manner of functional resonance. The final step is to propose ways of managing or diminishing the possible occurrence of uncontrolled performance variability.

The five-step approach of the FRAM can serve a number of purposes. The aim in the present study was to use the method to gain a detailed understanding of the functioning of hospital discharge among elderly patients. This paper thus applies the first three steps of the FRAM—identifying essential discharge functions, variability, and PSFs—which may account for variability and different outcomes across discharge practices.

An important consideration with the FRAM is determining the basis or categorization of successful functioning. With the FRAM, it is proposed that this categorization be developed based on a mutual understanding among a team of experts consisting of the people performing the functions under consideration [14]. However, given the broad range of stakeholders involved in hospital discharge, e.g., various health-care providers in different hospitals and primary-care units, patients, and their next of kin, this approach appears to be inadequate. We therefore define the concept of successful discharge functioning in terms of the perception of the stakeholders.

## Methods

### Study design and method

The observational case study approach of the present investigation was appropriate for examining local systems and organizational processes [15]. The use of observational research in conjunction with ethnographic research methods allows for a close analysis of naturally occurring social processes and practices within a given organizational context [16]. It offers the possibility of in-depth analysis within particular cases and theoretical generalizations among different cases. It can also allow a study of patterns of hospital discharge practices with different patients, thereby enabling the identification of functions, variability, and components affecting performance and outcome.

### Setting

The Norwegian health-care system comprises two organizational structures: primary care (general practitioner, nursing home, and home care); and specialized secondary care. Primary care is managed by local municipalities, whereas specialized health care is provided in state-owned hospitals and operated by four regional health authorities. The Coordination Reform [17] was implemented in January 2012. One of the main focal areas of the reform is to stimulate a good patient flow between hospitals and primary care institutions and to overcome challenges with delayed discharge better known as “bed blocking” (i.e., patient blocking beds in specialist care while awaiting municipal services) [18]. Several measures have been initiated to accomplish this goal, including legislation, mandatory agreements on cooperation between hospitals and municipalities, offering guidance, and providing financial incentives. The most important types of financial support are municipal cofinancing of specialist health-care services and municipal financial responsibility for patients who are ready to be discharged. Specifically, payment (fee of 533 euros per day) to an acute provider unit is required if the municipality does not accept the patient before midnight on the day they are deemed ready for discharge. Under the terms of the Coordination reform, hospital and municipalities are obliged to enter into legally binding contracts that set out formal requirements for care transitions and discharge planning [17].

### Sample and selection

The research was conducted in two hospitals in Norway (one rural and one city-based hospital) situated within the same regional health authority. The observations took place in three types of wards (geriatric, medical, and orthopedic) with the intention of developing cross-case comparisons of activity patterns across settings and among different patient groups. Seven wards were included in the study: one geriatric, three orthopedic, and three medical wards. In those seven wards, we selected 20 elderly patients (>75 years old) with orthopedic and medical conditions who would be in need of municipal services after discharge, e.g., rehabilitation, nursing home care, and home health care. To provide a comprehensive insight into hospital discharge practices, we sought to sample across the broad range of stakeholders involved in the process, i.e., health-care personnel groups, patients, and their next of kin. The broad inclusion criteria applied in this study (different hospitals, wards, patient groups, stakeholder perspectives) was intended to maximize data variation [19]. Table 1 presents the distribution of ward types, hospital types, and numbers of patients.

**Table 1:** **Distribution of patients in hospitals and wards**

Hospital (rural)	Patients	Hours of observation
Orthopedic ward	2	10 h
Medical ward*	4	20 h
**Hospital (city)**	**Patients**	**Hours of observation**
Orthopedic ward 1	2	11 h
Orthopedic ward 2	3	13 h
Specialized medical ward 1 (pulmonary diseases)	3	12 h
Specialized medical ward 2 (kidney diseases and infections)	2	9 h
Geriatric ward	4	15 h

*There was no specialized geriatric ward at the rural hospital.

### Data collection

We investigated the discharge of the 20 elderly patients; in line with the requirements of the FRAM approach, using moderate participant observations [20]. Moderate participant observation entails that the researcher be present and identifiable, though not an active participant (i.e., does not have a role in the social setting); the researcher observes and interacts occasionally. This type of participant observation allows the researcher to obtain a high level of involvement while maintaining a level of detachment [20].

The data were collected from March to October 2012. The data consisted of 90 hours of observation, including 173 conversations with patients, their next of kin, and health-care providers involved in the discharge processes. The first author (nursing background) conducted the observations based on a semi-structured observation guide, which is in accordance with the FRAM approach. The guide included topics that are relevant to hospital discharge, e.g., coordination, multi-disciplinary approach, information exchange, patient and family involvement, discharge planning, and challenges or barriers. In addition, the guide allowed for issues to emerge from the observations. In this way, it was intended to provide a detailed description of how discharge works. Observations started in the morning of the day of expected discharge, and they focused on interaction, coordination, and dialogue among health-care personnel and patients. During the observations, the researcher was dressed in hospital clothing so as to be inconspicuous [21].

Conversations with health-care personnel and patients were carried out in situ to clarify work practices and obtain assessments and viewpoints regarding the current discharge process [20]. The purpose of the conversations was also to stimulate dialogue about impressions and interpretations. Conversations with next of kin were conducted via telephone after discharge. Patients and next of kin were asked to describe their experiences of the discharge process. These conversations followed a particular structure. Besides being requested to relate experiences connected with the overall discharge process, stakeholders were asked about their satisfaction, involvement, participation, and concerns as well as information exchange, discharge planning, and possible improvements.

Copies of discharge summaries (with person-identifiable information deleted) were collected so that community-based health-care personnel could be asked to assess the written documentation and evaluate the overall quality of the current discharge process. No recording was made of the conversations owing to ethical considerations (confidentiality issues) and to the fact that numerous health-care providers, patients, and next of kin were involved (sound recording issues). Observation notes were written during the observations, and a summary of each, including researcher reflections opinions, was written immediately afterward.

### Data analysis

In line with the FRAM, data analysis involved a two-stage process: first, we identified common functions in the discharge process; second, we determined variability and PSFs within those functions. We identified common functions through an iterative process. All observational materials (150 written pages of field note summaries) were thoroughly reviewed individually by the first and second authors and then within a team of four researchers involved in the project (an experienced team with backgrounds in nursing, safety, user involvement, and change management). The functions were revised several times until final consensus was reached. A detailed description of the functions (including associated aspects—time, control, input, output, resources, and preconditions) was then developed based on an aggregated analysis of the 20 patient discharge cases, including the conversations with health-care personnel, patients, and their next of kin. Legally binding contracts (i.e., requirements for organizing hospital discharge) for the hospitals and municipalities included in this study were also used to support the description of functions.

The aggregated description of the functions and associated aspects was used for the analysis of variability and PSFs. The variability of each singular function was examined based on the descriptors from the various cases as well as the conversations with health-care personnel, patients, and relatives. Three analytic themes emerged through this process, which characterized the functional variability among the 20 patients: timing, duration, and precision in performance. PSFs were elaborated in the final step of the analysis using the aggregated description of the functions and associated aspects compared across the patient cases. The appropriate level of analysis at which to operationalize variety in organizational work processes has been questioned [22]. In the present study, PSFs were analyzed by applying a multilevel approach based on a stratification similar to Moray’s organizing framework of sociotechnical systems [23]. This entails that the analysis of PSFs involved the individual and team level as well as the organizational and contextual factors that were observed and expressed as being important. In the Results section, the main sources of variability among the patients are examined on an aggregated level, with the focus on general patterns.

### Ethical considerations

Ethical approval was obtained from the Committee for Medical and Health Ethics of Norway (REC, no. 2011/1978). This study was based on informed, voluntary consent among the patients, their next of kin, and health-care personnel. Ethical issues related to consent capacity were taken into consideration during the recruitment process. Recruitment during hospitalization may be ethically challenging owing to the ability of elderly patients to provide informed consent as a result of functional decline, strain, and cognitive impairments. The health-care providers at the hospitals assessed the cognitive functioning and overall situation of the patients and judged them as being suitable for recruitment. The researchers did not contact patients before they had provided their verbal consent to be contacted and informed about the study. Next of kin were included only if the patient approved of such contact. Next of kin were contacted by phone and informed about the study. The paper follows the STROBE guidelines for reporting of observational studies. An additional file shows the completed STROBE checklist [see Additional file 1].

## Results

### Hospital discharge functions

Hospital discharge takes place on a day-to-day basis, and involves complex, interdependent functions that require interaction and coordination among a multidisciplinary team of stakeholders, i.e., doctors, nurses, receiving health-care providers, patients, next of kin, and patient coordinators. This study identified 10 common functions that constitute the daily routine for discharging elderly patients from the hospital to primary health-care services in the municipality. The set of functions represent essential activities necessary for hospital discharge to succeed. The set of functions involve;

- Review of hospital inpatients—classifying patients that are medically fit for discharge
- Notifying the municipality that the patient is medically fit
- Informing the patient that they are ready for discharge
- Assigning an appropriate post-discharge site of care and notifying the hospital
- Notifying and informing the patient’s next of kin (if any)
- Preparing a nursing discharge record
- Preparing a medical discharge letter
- Providing oral information about the transfer to post-discharge care providers
- Ordering transportation
- Transferring the patient to the post-discharge site of care and ensuring the transfer of written information

A brief description of the identified functions, including a description of the essential associated aspects, is presented in Table 2.

**Table 2:** **Brief description of hospital discharge functions**

Function	Brief functional description	Contribution
Review of hospital inpatients—classifying patients that are medically fit for discharge.	Normally, hospital discharge is initiated by conducting a pre-ward round. The activity involves a clinical process in which the clinical care of hospital inpatients is reviewed. The responsible doctor reviews the patient’s progress and determines whether the patient is medically fit for discharge. The activity normally involves knowledge sharing among a multidisciplinary team, including the lead consultant, interns, junior doctors, responsible nurse (primary nurse or team nurse depending on the nursing care model applied at the ward), and sometimes physiotherapists. It is essential that all relevant information is shared to support the appropriate care decisions; this indicates that input is needed from multiple sources (i.e., information about the patient’s medical records, lab results, test results, medications, and functional and cognitive status). This function is controlled by guidelines for assessment stated in the regulations on municipal co-funding of patients ready for discharge [24].	Activates the discharge process.
Notifying the municipality that the patient is medically fit.	When the lead consultant has classified the patient as being ready for discharge, a message is sent to the receiving municipality (electronically, by phone or fax). For this notification to be considered valid, certain preconditions concerning discharge planning must be fulfilled as agreed upon in the cooperation agreements between the hospitals and municipalities.	Activates the discharge process in the receiving municipality; assigns an appropriate post-discharge site of care.
Informing the patient that they are ready for discharge.	The patient is normally informed about the decision for medical fitness during the ward rounds, which are the daily formal opportunity for dialogue and interaction among the patient, doctor, and care team. From the patient’s perspective (preparedness and satisfaction), it is essential that they have been prepared and involved in the discharge planning process prior to the day of discharge (to reduce anxiety, distress, and strain). The ward round normally takes place after the pre-ward round activity is completed, and it is conducted at the patient’s bedside. Normally, several professionals attend the ward rounds. In general, the round is led by the senior doctor or doctor in charge of the ward, with junior doctors or medical students and nursing staff present. This function is controlled by regulations stating the patient’s right to information, participation, and involvement [25].	Prepares and provides the patient with discharge information or instructions and plans for follow-up care.
Assigning an appropriate post-discharge site of care and notifying the hospital.	The receiving municipality has (according to the cooperation agreement) a 3-hour response time (from the time the notification of the patient being medically fit is received—if sent correctly) to contact the hospital and indicate whether and when a post-discharge site of care is available. For the municipality to determine the most appropriate setting for post-discharge care, it is essential that there is compliance with the discharge planning agreements and that the hospital provides accurate and sufficient information. Different ways of organizing the coordination in discharge planning are recognized depending on the municipality size. In a city region, patient coordinators in the municipality are responsible for organizing the information exchange during the discharge. In a rural region, a helpline has been established across municipalities with an assigned person (i.e., head nurse at a nursing home) responsible for coordination in each municipality. In the city region, information is exchanged electronically between the hospital and patient coordinators in the municipality; in the rural region, this is done over the phone or via fax.	Avoids delayed discharges. Determines the most suitable post-discharge site or level of care.
Notifying and informing the patient’s next of kin (if any).	Normally, the patient’s nurse contacts (usually over the phone) the patient’s next of kin (if any) to inform them about the discharge and plans for follow-up care when clarified. From the next of kin’s perspective, it is essential that they are provided with information and are involved in the discharge planning process prior to the day of discharge. This function is controlled by regulations stating that the patient’s next of kin should receive information about the patient’s state of health, treatment, and care provided (if the patient has given their consent) [25].	Prepares and provides the patient’s next of kin with discharge information and plans for follow-up care.
Preparing a nursing discharge record.	The nursing discharge record is completed according to statutory regulations [26], stating that the patient’s record shall be sent to the professionals who need the information to provide the patient with appropriate follow-up care. The nursing record should include descriptions of the nursing care delivered, the patient’s status, assessments, and recommendations for continuing care.	Ensures written information transfer and continuity of care.
Preparing a medical discharge letter.	The medical discharge letter is similarly governed by regulations [26], stating that the discharge summary must contain information about the patient’s medical diagnosis and former medical history, treatment performed during hospitalization, functional level and assessment, a complete medical list, and prescriptions for new medications. Plans for follow-up care are also provided. The nursing and medical record is normally not prepared until after the patient is deemed medically fit for discharge.	Ensures written information transfer and continuity of care.
Providing oral information about the transfer to post-discharge care providers.	When post-discharge arrangements have been clarified and confirmed by the receiving municipality, the patient’s nurse contacts the assigned care facility to provide direct oral information about the patient. The function depends on pre-conditions, such as information and knowledge about the patient, follow-up care plans, hospital course, treatment, and current medications. The latter is emphasized as important to ensure that the receiving care providers or site of care have the patient’s current medications available.	Ensures the continuity of care and agrees on a time of transfer.
Ordering transportation.	Transportation can be arranged and ordered after it has been clarified when and where the receiving municipality has availability. Patients can either be transported to the post-discharge site of care by ambulance, by taxi, or by next of kin, according to their conditions and preferences. If an ambulance is required, an order is sent electronically, which also specifies the time the patient will be ready for transfer.	Arranges suitable transportation.
Transferring the patient to the post-discharge site of care and ensuring the transfer of written information.	To ensure the continuity of care, it is crucial that written information be present and available when the patient leaves the hospital. This function is controlled by regulations [26], the cooperation agreement, and by established routines or procedures at the wards, which state the information that is to be provided. The information (nursing record and discharge letter) is sent with the patient on discharge in addition to being sent electronically or by post (to the receiving care providers and the patient’s general practitioner). From the perspective of those assuming responsibility for post-discharge care, it is desirable that the patient be transferred and arrives during the daytime (9 a.m. to 3 p.m.) since more resources and greater competence are available then. There is also a challenge for health-care personnel in the municipality to contact hospital staff for clarification if the responsible doctor or nurse has ended their shift and the next shift has little knowledge of the patient. Similarly, hospital personnel prefer to transfer patients that are ready for discharge during the day shift to safeguard the process and avoid shift handover issues.	Ensures the continuity of care. Ends the hospital discharge process: the patient physically leaves the hospital, and the municipality takes over responsibility.

The functional descriptions provided in Table 2 demonstrate that hospital discharge is a complex multi-agency care process, which is composed of multi-functional activities aimed at accomplishing many goals. Those goals include making appropriate care decisions, assigning an appropriate post-discharge site of care, avoiding delays in the discharge, transfer information, continuity of care, and the preparation and involvement of patients and their next of kin. On an aggregated level, the set of functions primarily involves decision-making and knowledge-sharing activities among various health-care personnel, disciplines, patients, and their next of kin. We need to examine how these functions vary in everyday discharge practices.

### Performance variability—observed functioning of discharge practices

Our data indicated substantial variability in the way discharge functions are performed. Accordingly, patients, their next of kin, and health-care personnel reported some discharge practices as having been more successful than others. Success here was defined in terms of reported quality of information transfer, the level of post-discharge care, the duration of the process, and level of satisfaction. We found considerable variability in the discharge functions in three main dimensions:

1. *Timing* (the time of day the discharge functions were carried out),
2. *Duration* (the time spent performing the functions) and,
3. *Precision* (performance characteristics and perceived success of the function by the various stakeholders)

We found time to be a main source of variability. The temporal range in the functional variability was the duration of the discharge process, and it varied considerably among the 20 patients, from a few hours to a few days. The main variations in precision were related to the following: (1) decision-making criteria with respect to medical fitness and post-discharge arrangements; (2) the quality of the discharge planning process; (3) patient participation and engagement of their next of kin; and (4) the quality of the information transfer. The variability for each function and the recognized and reported outcome variability are presented in Table 3.

**Table 3:** **Functional performance and outcome variability in hospital discharge of elderly patients**

Functions	Variability in discharge practices		Variability in outcome
	Time and duration	Precision	
Review of hospital inpatients—classifying patients that are medically fit for discharge.	Time of day when the decision was determined.	Criteria upon which the decision was established and degree of knowledge sharing with the care team.	Time of day the patient was determined medically fit (i.e., duration of the discharge process) Patient’s degree of readiness Receiving health-care provider’s degree of satisfaction with the decision about medical fitness.
Notifying the municipality that the patient is medically fit.	Time of day when the municipality was notified.	Degree of compliance with cooperation agreements.	Duration of the discharge process (i.e., delay in the discharge process in the case of non-compliance).
Informing the patient that they are ready for discharge.	Time of day the patient was informed and time allotted to each patient.	Approaches concerning content or type of information provided, the language used, and how the patient was approached.	Patient involvement in the discharge planning process and degree of satisfaction.
Assigning an appropriate post-discharge site of care and notifying the hospital.	Time of day the hospital was notified	Criteria for prioritizing patients for post-discharge care.	Duration (i.e., number of additional days spent after being determined medically fit). Level of post-discharge care offered. Degree of satisfaction concerning post-discharge arrangements.
Notifying and informing the patient’s next of kin (if any).	Time of day relatives were informed and time spent.	Degree of information provided and by whom (level of competence, doctor or nurse).	Next of kin’s degree of satisfaction and perceived involvement in the discharge planning process.
Preparing a nursing discharge record.	Time of day the record was prepared and time available (time spent).	Prevalence and quality of the contents.	Quality of the information transfer Receiving health-care provider’s degree of satisfaction.
Preparing a medical discharge letter.	Time of day the letter was prepared and time available (time spent).	Quality of the contents, structure, and readability.	Quality of the information transfer Receiving health-care provider’s degree of satisfaction.
Providing oral information about the transfer to post-discharge care providers.	Time of day and time spent.	Degree and quality of the information provided and by whom (level of competence).	Receiving health-care provider’s degree of satisfaction.
Ordering transportation.	Time/hour arranged for transfer.	The choice of arrangements and transportation (taxi, ambulance, next of kin) and the dialogue between the doctor and nurse.	The responsible doctor’s involvement in the decision concerning the time for transfer—affected degree of time pressure to prepare the medical discharge letter.
Transferring the patient to the post-discharge site of care and ensuring the transfer of written information.	Time of day the patient was transferred.	Degree of compliance with arrangements. Unpredictable if carried out by the ambulance service (owing to simultaneous responsibilities).	Time of day the patient arrived in primary care and the receiving health-care provider’s degree of satisfaction with the time of arrival.

### Performance-shaping factors

A PSF is anything that affects the health-care provider’s performance of a function within the health-care system [27]. We found multiple, diverse PSFs, which accounted for the variability presented in Table 3. In this section, we will examine only the main variations.

### Temporal conditions

Temporal variability across the observed cases was typically determined by the three functions indicated below. These functions served either to activate or delay the discharge process, and they thereby influenced the overall duration of the discharge processes (from being determined medically fit to the transfer of care). Variability in these three linked functions created time constraints on associated functions. The three functions were as follows:

- Review of hospital inpatients—classifying patients that are medically fit for discharge.
- Notifying the municipality that the patient is medically fit.
- Assigning an appropriate post-discharge site of care and notifying the hospital that site.

One of the most critical functions is the review of hospital inpatients to determine whether a patient is medically fit for discharge. This function activates the overall discharge process and affects all subsequent functions by determining when they are initiated. Considerable variations were identified in terms of the actual time (hour of day) the patient was determined medically fit; the range was from 9 a.m. to 1:30 p.m. The discharge process was found to be more rushed when the patients were declared medically fit later in this period, i.e., after noon. This was because of the reduced possibility to prepare the discharge requirements for care transfer if the transfer was to take place the same day. The health-care personnel clearly stated that time pressure potentially increased performance variability, affecting precision issues. The following statements reflect these concerns:

- “It’s busy . . . of course there is an increased chance or risk that you forget something.” (Chief doctor, orthopedic ward)
- “It’s clear that things can happen a lot faster toward the end of the day.” (Head nurse, orthopedic ward)
- “After the decision was made that I was ready to be discharged, it was a rush right up to the time I left . . . It was like I had to get dressed and get out.” (Patient, female 87 years)

Other factors stated as influencing the duration were as follows: the quality of the discharge planning process; patient characteristics; the degree of simultaneous responsibilities among the clinical team; the degree of familiarity with the inpatients; and the availability of sufficient resources, i.e. updated patient information. Doctors often referred to pending lab and test results as a factor that guided the decision about medical fitness; this affected the duration and completeness of the decision-making process.

The temporal completeness of the decision about medical fitness determined the time (hour of day) of notifying the local municipality. This function activates the discharge process for the receiving municipality (i.e., assigning an appropriate post-discharge site of care); if there are delays through late notification, this puts time pressure on the municipality personnel. Bed availability in the receiving municipality determined whether the patient was assigned a post-discharge site of care on the day they were determined medically fit or if they had to spend additional days in the hospital—a delay in the discharge process.

Time variations in determining medical fitness have knock-on effects across the system. In particular, when decisions are made later in the day, this created time pressure for local municipality personnel, who had to initiate the functions related to care planning and post-care transfer. This time pressure was exacerbated by financial penalties for delayed discharge; these encourage municipal staff to rush care planning to avoid paying the daily fee. Such time pressure could have a knock-on effect in terms of the precision of care planning. Only five of the 20 patients in our study spent additional days at the hospital: the time varied from 1 to 5 days. It was emphasized, especially by nurses, that there was better time to prepare and perform the discharge functions if the patient spent additional days at the hospital. Some nurses acknowledged that the available time could affect precision issues in particular related to patient and next of kin involvement, discharge planning, and quality of information transfer. This was confirmed by the patients and their next of kin. Patients who spent additional days in the hospital stated that they had more time to prepare mentally for the discharge, and this appeared to be connected with a higher level of patient and next-of-kin satisfaction.

### Precision issues

We identified considerable variability in the decision-making criteria concerning both the decision about medical fitness and post-discharge arrangements. The quality of the discharge planning process also varied among the patients, as did patient involvement and quality of information transfer. As indicated above, the temporal conditions (i.e., degree of time pressure) are a major PSF that influences the precision issues. Below, we describe other PSFs that influence precision.

#### Medically fit for discharge

There was variability in the criteria for the decision about medical fitness and its quality, especially among the hip fracture patients. For example, doctors appeared to put different emphasis on involving and consulting with the responsible nurse or the patient in their decision-making process. At one ward (orthopedic), nurses were not present when the decision for medical fitness was determined. This was explained as being due to institutionalized routine and effectiveness. The contribution of nurses to the decision-making process varied. Some were passive and did not interact with the doctor; others participated more actively. Work experience, relationship with the doctor, the doctor’s characteristics, and the degree of familiarity with the patient were cited as possible explanatory factors for this. In several cases, doctors and nurses were unfamiliar with the patient; this was explained as being due to time off work, the patient’s short hospital stay, and high patient turnover. The degree of familiarity (i.e., care continuity) was observed to affect the level of knowledge sharing among the doctors and nurses in the decision-making process. Some orthopedic doctors also indicated that they were more thorough (spent more time) with patients for whom they felt responsible (e.g., patients on whom they had performed surgery), and this potentially influenced the decision-making process.

Nurses in municipal services experienced variability in the doctors’ criteria for regarding patients as medically fit. Some wards seemed to pay more attention to the patients’ overall health-care status rather than the strict clinical condition; this was particularly true of hip fracture patients. For example, there was a lack of attention to the underlying social or physical causes of a patient’s fall and hip fracture. In contrast, geriatric wards appeared to make more holistic decisions and took into account factors beyond the medical determinants, such as assessing activities of daily living, cognition, social support, psychological well-being, and psychosocial factors.

Another factor that affected the decision-making criteria was bed availability. Doctors were particularly under pressure to discharge patients when units were crowded. In one case, it was observed that the head of a medical department on a morning visit informed the head nurse that they should discharge patients that day since there had been many new arrivals in the emergency unit.

#### Post-discharge arrangements

The level and site of post-discharge care varied among the patients; these especially affected the patient and next-of-kin satisfaction with the discharge process. The next of kin appeared to be more pleased if the patient was discharged to a nursing home rather than to home with health-care services. The majority of the patients in this study were discharged to a higher level of post-discharge care than the care they had received prior to admission. Of the 20 patients, 18 were admitted to the hospital from home; of these, 16 were discharged for a short-time stay at a nursing home. The remaining four patients were discharged directly to home with home health-care services. Not all the patients in this study had next of kin; however, for those that did, the next of kin played an important role as advocates in the decision making. In some cases, the next of kin questioned whether their involvement and persistence had an impact on the level of post-discharge care offered.

According to the patient coordinators (responsible for determining the appropriate level of post-discharge care), a number of factors influenced the decision-making process. These factors included the following: information and recommendations provided by the hospital; the quality of the discharge-planning process; prior knowledge or familiarity with the patient (the nature of the patient’s current home and its suitability for the patient’s condition and the presence of next of kin); degree of pressure from the next of kin; financial incentives; and current availability of beds and resources in the receiving municipality. According to hospital providers, some municipalities struggled more with availability than others. For some patients, the patient coordinator and assigned municipality staff tried to negotiate a later discharge date if the municipality had limited capacity. Hospital providers also stated that owing to a lack of bed availability and to avoid paying the daily fee, municipalities could decide to discharge patients directly to home with home-based nursing care—even if a short-term nursing home stay was recommended by the hospital.

#### Quality of the discharge planning process

The degree of compliance with discharge planning agreements varied among the patients. Municipality personnel, i.e., patient coordinators and assigned contact people, stressed the importance of good discharge planning (compliance with discharge arrangements and close dialogue during the hospital course) in determining the most appropriate setting for post-discharge care and to avoid delays. Short hospital stays were a challenge in the discharge planning process. For example, it was not unusual for municipality personnel to receive the medical and nursing records describing the patient’s activity level and cognitive status on the same day as they received the decision for medical fitness. As such, patient coordinators had less time to make the preparations for the necessary post-discharge care. It was also apparent that some units were more efficient than others in notifying the municipality early on the day of discharge. Ward leadership seemed to play a key role in this regard. Some head nurses were more active in communicating with the care team. They verified compliance with the discharge planning agreements and reminded the responsible nurses to send notification about the patient being medically fit for discharge to the municipality.

#### Degree of patient participation and engagement of next of kin

This study also found considerable variability in how patients and their relatives were involved in the discharge planning process; this influenced the success of the post-discharge planning and overall satisfaction. The notification about discharge was often unexpected, which indicates that patients and their next of kin may have been insufficiently involved in the discharge planning process.

New discharge planning demands (i.e., requirements for information exchange, notifications during hospitalization) increased administrative work, and documentation limited the interaction between health-care providers (especially nurses) and patients and their next of kin. We found that the interaction between health-care personnel and patients varied according to the following: the type of information provided; the language used; how the personnel approached the patient (standing, speaking above the patient, speaking directly to the patient); the engagement with patient preferences; and different degree of encouragement.

The time allotted to each patient when providing the discharge information also varied considerably—from 3 to 10 minutes. Doctors and nurses offered several explanations for this variability, such as individual characteristics and communication skills, patient characteristics, various conditions and preferences for participation, and time pressure. The patient’s characteristics (cognitive or mental status, disabilities, communication skills, and complexity of care) and preferences also showed great variation. Some patients were more active than others or had more knowledge of their situation, diagnoses, and medications; this appeared to affect the degree of information and knowledge sharing between patients and doctors.

#### Quality of the information transfer

The quality of the information transfer, i.e., nursing records and medical discharge summaries, was reported to vary considerably among the patients. A nursing record was present for 16 of the 20 observed patients, and a medical discharge letter was available for all the observed patients. With 11 patients, health personnel outside the hospital reported information inconsistencies or inaccuracies, such as missing information about medicine regimes, lab test results, or follow-up care plans. For three patients, the medication list was lacking (hip fracture patients), and two patients were discharged with the wrong medication list.

The receiving health-care providers generally found that the discharge letters prepared by the medical doctors, especially geriatric doctors, were of good quality; conversely, the surgical discharge summaries tended to have more incomplete or missing information. For three patients, there was inconsistency between what the nurse wrote and what the doctor wrote about the same patient. Variability in the content and quality here may be explained by the character of the hospital unit and the doctor’s specialization and preferences. Deficiencies in the nursing records at discharge were explained by the hospital nurses as being due to the lack of care continuity, a short hospital stay, insufficient and poor documentation, temporal conditions, and the degree of simultaneous responsibilities (e.g., number of patient discharges for which the nurse was responsible, new admissions requiring attention). Information provided in nursing records was often characterized by cutting and pasting from previous documents if nurses were unfamiliar with the patient.

Senior doctors often delegated the responsibility for preparing the medical discharge summary to interns, junior doctors, or medical students. However, we found variations in the senior doctors’ quality assessment of records before being sent with the patient or to receiving health-care personnel. At some hospital wards, it was a standard procedure for all discharge letters to be approved by a senior doctor, but in others this was not normal practice. Hospital doctors referred to several influential factors that affected both temporal and precision issues. These included the following: level of familiarity with the patient; degree of available and accurate information; patient characteristics (e.g., degree of complexity of condition, length of hospital stay); information input overload (influenced by the patient characteristics, length of hospital stay, number of transfers within the hospital, number of doctors involved); time pressure (influenced by the time of day the letter was prepared); and the degree of simultaneous responsibilities (e.g., the number of patient discharges, new admissions requiring attention, and other tasks to perform). Nurses at the orthopedic wards reported inconsistencies and unpredictable patterns related to the doctors’ presence in the wards, which resulted in difficulties in preparing a medical discharge document on time.

In summary, this study identified multiple, diverse PSFs that influenced the functioning of hospital discharge. They included the following: variations attributed to temporal conditions (i.e., degree of time pressure) surrounding the discharge process; the characteristics of the individuals and care team involved (doctors, nurses, other members of the care team and their approach, preferences, risk awareness, decision-making criteria, communication and team skills); variability in patient factors (i.e., resources, preferences, cognitive or mental status, disabilities, communication skills, complexity of care); organizational factors (i.e., the unit, specialization, work organization, leadership, institutionalized routines); and local work environment factors (i.e., bed availability, familiarity with the patient, current availability in municipal services, simultaneous responsibilities, quality of the discharge planning process, and degree of pressure from the next of kin).

## Discussion

Most research about hospital discharge has tended to focus on particular, isolated aspects (i.e., information transfer, discharge planning, patient participation, medication reconciliation) [28–31], specific outcome measures (i.e., adverse events, readmission rates, adverse drug events, satisfaction with care) [32–36], or the experiences of professional groups or stakeholders in isolation [10, 28, 37]. As such, the present study is unique since it applies a multiple-stakeholder perspective in examining hospital discharge functions, variability and the factors contributing to the variability, and perceived outcomes in discharge practice. Through the application of the FRAM, this study expands our understanding about the complexity of hospital discharge and context-specific factors that explain hospital discharge, shape performance, and introduce variability.

This paper demonstrates that the FRAM is a powerful method for studying and analyzing the complexity of hospital discharge practice; it provides a detailed, systemic analysis of hospital discharge for elderly patients, which has not previously been presented. Our findings illustrate how hospital discharge for elderly patients is a commonly occurring function, though it varies in numerable ways. By observing the everyday practice of hospital discharge for these patients, we have identified the common functions that typically occur on the day of discharge and the multiple, diverse sources of performance variability among those functions (i.e., timing, duration and precision issues).

Individual characteristics are an important determinant of performance [38], and studies conducted of PSFs in health care at the individual level have largely focused on fatigue, stress, and aging [39]. The interaction of individual characteristics is fundamental to team performance [40]. The present study emphasizes the importance of knowledge sharing, especially among doctors and nurses, toward appropriate decision making. The degree of familiarity with the patient was perceived to have strong implications for the quality and level of knowledge sharing among the members of the care team. This is in accordance with previous findings, where a lack of familiarity with patients was found to compromise assessments and the decision- making process [10]. Research on team performance has been conducted within specific settings, especially in intensive care units, operating theaters, and emergency medicine, and has been largely concerned with emergency patient-care processes [41]. Less attention has been given to the role of team performance on more complex inter-organizational processes, such as hospital discharge. This area needs to be investigated further along with factors that facilitate or constrain successful team performance during hospital discharge.

Individual and team performance is further influenced by organizational factors, e.g., unit, specialization, leadership, work organization, and institutionalized routines. Hospital wards are highly specialized and are perceived as shaping the clinician’s and care team’s preferences, attention, information exchange, and decision-making criteria. In this study, the unit of analysis (the hospital ward) had an impact on outcome (i.e., satisfaction, decision-making criteria, and quality of information transfer). The receiving health-care care providers appeared to make more negative remarks about the process related to patients discharged from orthopedic wards than from medical, especially geriatric, wards. The importance of geriatric knowledge and assessment has been investigated in previous studies [10, 42, 43]; there, it was argued that increasing specialization within health professions and fragmentation through disciplinary knowledge may result in inappropriate decisions that fail to meet the complex needs of patients [10, 40]. Despite such concerns, the impact and effect of organizational factors (e.g., ward specialization) related to specific discharge processes and outcomes demands investigation. Future studies should extend our understanding of the relationship among ward or clinical specialization, discharge functioning, and discharge outcomes.

Our findings also raise the awareness of the temporal aspects related to the current discharge processes. The results of this study strongly suggest that the time of day the patient is declared medically fit is important: this determines the temporal conditions (degree of time pressure) for the subsequent actions. This decision about medical fitness being made later in the day (after noon) was associated with increased time pressure; it led to variability, and it affected duration and precision issues. Previous studies have addressed the importance of the timing of discharge [10]; they indicated that the time interval (i.e., time between making the decision for a patient to be discharged and the actual transfer) is a potential barrier for information sharing since time constraints lead to less flexibility, greater time pressure, and increased performance demands [44]. Psychological studies have shown that time pressure decreases performance standards [45]. However, this matter has not been systematically addressed within health care.

A key contextual factor that was perceived as affecting the temporal completeness of the decision about medical fitness was availability of beds. The problem of crowded wards and its implications on performance have been illustrated with the notion of “going solid” [46], and it leads to increased pressure to discharge patients so as to make way for new ones. It puts pressure on the clinical decision-making process, encouraging staff to accelerate the completion of care, increases performance pressure, and creates the potential for poor performance [46]. Our results suggest that the time aspects influencing discharge performance and outcome should be further examined for hospital discharge practices on a larger scale.

The results presented here further emphasize the role of the elderly patient (i.e., their resources, preferences, needs, communication skills, cognitive and functional status, and capacity to participate) and that of their next of kin (i.e., preferences, involvement, and degree of pressure) in the reported satisfaction with the discharge process and outcome. Patient factors have been found to affect the elderly patient’s ability to be involved or participate in the discharge process [47]; however, knowledge is limited on the factors that facilitate or hinder patient-centered performance during the discharge process [48]. It has been suggested that clinicians should put more effort into understanding patients’ and relatives’ preferences for participating in decisions concerning discharge and that clinicians should tailor their approach to meet specific needs [49]. The time allotted, language used, number of people present, and disturbing elements were described as factors that influenced patients’ and relatives’ involvement, understanding, and level of satisfaction. In accordance with our results, financial factors, lack of familiarity with the patient, bed availability, and lack of time have been identified as factors that constrain patient-centered performance [44, 48].

With the inclusion of the multiple-stakeholder perspective, our findings also reveal one of the main challenges with the FRAM approach in the context of health-care delivery. The FRAM appears to emphasize health-care providers’ definitions and concepts of acceptable, successful outcomes without considering the experiences of patients and their next of kin. Our results illustrate that the various stakeholders had different concerns and used different measures to evaluate the degree of successful hospital discharge functioning. This study implies that the assessment of acceptable, successful outcomes depends on the focus of the stakeholder groups. We argue that the process of determining successful outcomes must incorporate all stakeholder groups. The multiple perspectives of all stakeholders, including patients and their next of kin, have not received systematic attention in the literature on hospital discharge [10]; it has been suggested that the experiences of patients and their next of kin provide valuable input and can help produce improvements [50–52].

From our results, we would argue that the multiple PSFs related to hospital discharge and multiple-stakeholder perspectives have not been fully considered in interventional studies targeted at improving this process. Our findings illustrate that it is insufficient to isolate functions (i.e., merely consider information transfer, patient participation, decision-making processes) as independent activities (i.e., treat them as “functional silos”) owing to the functional dependencies on which hospital discharge performance relies. Future studies on hospital discharge should consider the health-care providers involved, the available resources, the patient being discharged, their next of kin, the organizational setting, and the current situational factors related to the functioning of discharge. Without considering these interdependencies, progress on hospital discharge improvements will be constrained [9, 14, 53, 54].

### Study limitations

There are several limitations that should be considered when interpreting our results. The observations took place during regular working hours (8 a.m. to 4 p.m.). Thus, evenings, nights, and weekends were excluded owing to practical and resource-based issues. This represents a possible limitation because other performance issues (variability, PSFs) may be influential at other times. This study was performed in the context of the Norwegian health-care system with a relatively small sample size (20 patients) in two hospitals, which restricts the generalizability of the findings. Possible observer bias should also be mentioned since the observations were conducted by a single researcher (first author) with a nursing background, which entails a pre-understanding of the context. Such an inside perspective may advance data collection but also affect the accuracy of the observations. An observation team with a minimum of two researchers with different backgrounds could better cover the complexity of the observation setting involving both professional and patient or next-of-kin perspectives. We tried to control this observer bias by setting up weekly meetings or updates in the observation periods with the larger research team (the members have backgrounds in nursing, management, and safety) to discuss preliminary impressions. Triangulation during the analysis process was carried out, with the three authors and members of the research team all being active in discussing the findings. Following the aims of the paper we have chosen to focus on the FRAM`s applicability to hospital discharge to explore its characteristics (e.g. functions) and general patterns of variability in discharge practices rather than addressing the specificities of each case. Finally, the study focused on the final stage of hospitalization, i.e., the actual discharge process. It would have been valuable for the study to have acquired data on the patients’ course from the day of admission to the end of their hospital stay.

## Conclusions

Hospital discharge is a complex multi-agency care process that is composed of multi-functional activities; it has multiple purposes, but its core activities are decision making and knowledge sharing. Through the application of the FRAM and use of observational methods, we have provided detailed insight into the range of functions that are performed during hospital discharge. We have called attention to the ways in which these functions vary, and we gained insight into the multiple PSFs that can be attributed to a range of contextual features (situational, organizational, individual teams, patients, next of kin, regulatory influences and interdependencies). Such multifaceted understanding of PSFs is necessary in improving hospital discharge practices.

Based on our findings, we argue that the existing, sequential approaches to the complexity of hospital discharge are inadequate. Given the interdependence among the functions, there is a need for corresponding multi-factorial interventions. Future research should focus on understanding the relationships between various functions and PSFs and their impact on hospital discharge practices and outcomes.

Study results illustrate that the FRAM represents a powerful methodology, enabling new insight into complex inter-organizational processes. Further on study findings emphasize that functional performance and outcomes entail various stakeholder perspectives whereby assessment of acceptable, successful performance and discharge outcomes depends on each individual perspective. These differences in outcome values need to be acknowledged in order to create a common ground on what constitutes acceptable, successful discharge functioning.

## Electronic supplementary material

Additional file 1: **STROBE Statement—checklist of items that should be included in reports of observational studies.** (DOC 88 KB)

## References

[1] Laugaland K, Aase K, Barach P, Albolini S, Bagnare S, Bellani T, Llaneza J, Rosal G, Tartaglia R (2011). Addressing risk factors for transitional care of the elderly – Literature review. Healthcare Systems Ergonomics and Patient safety 2011 – An alliance between Professionals and Citizens for Patient Safety and Quality of Life.

[2] Foster AJ, Murff HJ, Peterson JF, Gandhi FJ, Bates WD (2003). The incidence and severity of adverse events affecting patients after discharge from the hospital. Ann Intern Med.

[3] Long JS, Brown FK, Ames D, Vincent C (2013). What is known about adverse events in older medical hospital inpatients? A systematic review of the literature. Int J Qual Health Care.

[4] Vincent C (2010). Patient Safety.

[5] Tsilimingras D, Bates DW (2008). Addressing postdischarge Adverse events: A Neglected Area. Jt Comm J Qual Patient Saf.

[6] Kripalani S, LeFevre F, Phillips CO: **Deficits in Communication and Information Transfer between Hospital- Based and primary care physicians.** *JAMA* 2007.,**297**(8)**:** [http://jama.jamanetwork.com/article.aspx?articleid=205790] 10.1001/jama.297.8.831 17327525

[7] Rennke S, Nguyen O, Shoeb H, Magan Y, Wachter R, Ranji RS (2013). Hospital- initiated Transitional Care interventions as a Patient safety Strategy. Ann Intern Med.

[8] Rowley E, Waring J (2011). A Socio-cultural perspective on patient safety.

[9] Abraham J, Kannampallil GT, Patel LV (2012). Bridging gaps in handoffs: A continuity based approach. J Biomed Informantics.

[10] Robinson CA, Bottorff JL, Lilly MB, Reid C, Abel S, Lo M, Cummings GG (2012). Stakeholder perspectives on transitions of nursing home residents to hospital emergency departments and back in two Canadian provinces. J Aging Stud.

[11] Hollnagel E (2013). An Application of the Functional Resonance Analysis Method (FRAM) to Risk Assessment of Organizational Change. Swedish Radiation Safety Authority, Report number.

[12] Rankin A, Lundberg J, Woltjer W, Rollenhagen C, Hollnagel E, Resilience in Everyday Operations (2013). A Framework for Analyzing Adaptions in High- Risk Work. J Cognitive Eng Decision Making.

[13] Amalberti R, Hollnagel E, Braithwaite J, Wears R (2013). Resilience and Safety in Health Care: Marriage or Divorce?. Resilient Health Care.

[14] Hollnagel E (2012). FRAM: the functional resonance analysis method.

[15] Yin RK (2003). Case study research: design and methods. Applied social research methods serie.

[16] Leslie M, Paradis E, Gropper AM, Reeves S, Kitto S (2013). Applying ethnography to the study of context in healthcare quality and safety. BMJ Qual Saf.

[17] *Report no. #47 (2008–2009). Report to Parliament: Coordination reform: Proper treatment – at the right place and right time*. [http://www.regjeringen.no/upload/HOD/Samhandling%20engelsk_PDFS.pdf]

[18] Majeed MU, Williams DT, Pollock R, Amir F, Liam M, Foong SK, Whitaker JC (2012). Delay in discharge and its impact on unnecessary hospital bed occupancy. BMC Health Services Res.

[19] Miles MB, Huberman MA (1994). Qualitative data analysis.

[20] Dewalt KM, DeWalt BR (2011). Participant observation: a guide for fieldworkers.

[21] Hammersley M, Atkinson P (2007). Ethnography: Principles in practice.

[22] Pentland TB (2003). Sequential Variety in Work Processes. Organizational Sci.

[23] Moray N (2000). Culture, politics and ergonomics. Ergonomics.

[24] Law concerning Pri (2012). Law concerning Primary Health and Care Services.

[25] *Patient right Act*. 1999. [http://lovdata.no/all/nl-19990702-063.html]

[26] *The Norwegian Patient Record Regulation*. 2000. [http://lovdata.no/dokument/SF/forskrift/2000-12-21-1385]

[27] Rooney JJ, Vanden Heuvel NL, Lorenzo KD, Reduce human error (2002). How to analyze near misses and sentinel events, determine root causes and implement corrective actions. Quality Progress. American Society for Quality.

[28] Hilligoss B, Cohen M (2013). The Unappreciated Challenges of Between- Unit Handoffs: Negotiating and Coordinating Across Boundaries. Ann Emerg Med.

[29] Bauer M, Fritzgerald L, Haesler E, Manfrin M (2009). Hospital discharge planning for frail older people and their family. Are we delivering best practice? A review of the evidence. J Clin Nurs.

[30] Foss C, Hofoss D, Romøren TI, Bragstad LK, Kirkevold M (2012). Elderly patients’ experiences with hospital discharge (in norwegian). Sykepleien Forskning.

[31] Vira T, Colquhoun M, Etchells E (2006). Reconcilable differences: correcting medication errors at hospital admission and discharge. Qual Saf Health Care.

[32] Foster AJ, Clark HD, Menard A, Dupuis N, Chernish R, Chandok N, Khan A, Van Walraven C (2004). Adverse events among medical patients after discharge from hospital. CMAJ.

[33] Mesteig M, Helbostad LJ, Sletvold O, Røsstad T, Saltvedt I (2010). Unwanted incidents during transition of geriatric patients from hospital to home: a prospective observational study. BMC Health Serv Res.

[34] Coleman EA, Smith JD, Raha D, Min SJ (2005). Posthospital medication discrepancies: prevalence and contributing factors. Arch Intern Med.

[35] Gruneir A, Dhalla AI, Walraven C, Fischer DH, Camocho X, Rochon AP, Anderson MG (2011). Unplanned readmissions after hospital discharge among patients identified as being at high risk for readmission using a validated predictive algorithm. Open Med.

[36] Bull MJ, Roberts J (2000). Components of a proper hospital discharge of elders. J Adv Nurs.

[37] Reid CR, Cummings EG, Cooper LS, Abel LS, Bissell JL, Estabrooks AC, Rowe HB, Wagg A, Norton GP, Ertel M, Cummings GG (2013). The Older Persons’ Transition in Care (OPTIC) study: pilot testing of the transition tracking tool. BMC Health Serv Res.

[38] Patterson SE, Woods DD, Roth ME, Cook IR, Wears R, Render LM (2006). Three Key Levers for Achieving Resilience in Medication Delivery with Information Technology. J Patient Safety.

[39] Leblanc RV, Manser T, Weinger BM, Musson D, Kutzin J, Howard KS (2011). The Study of Factors Affecting Human and Systems performance in Healthcare Using Simulation. Simul Healthc.

[40] Nancarrow AS, Booth A, Ariss S, Smith T, Enderby P, Roots A (2013). Ten principles of good interdisciplinary team work. Hum Resour Health.

[41] Manser T (2009). Teamwork and patient safety in dynamic domains of healthcare: a review of the literature. Acta Anaesthesiol Scand.

[42] Kleinpell RM, Fletcher K, Jennings MB, Hughes RG (2008). Reducing Functional Decline in Hospitalized Elderly in Patient Safety and Quality. An Evidence-Based Handbook for Nurses.

[43] Caplan GA, Williams AJ, Daly B, Abraham K (2004). A randomized, controlled trial of comprehensive geriatric assessment and multidisciplinary interventions after discharge of elderly from the emergency department – the DEED II study. J Am Geriatrics Soc.

[44] Glenny C, Stolee P, Sheiban L, Jagal S (2013). Communicating during care transitions for older hip fracture patients: family caregiver and health care provider’s perspective. Int J Integrated Care.

[45] Tsiga E, Panagopoulou E, Sevdalis N, Montgomery A, Benos A: **The influence of time pressure on adherence to guidelines in primary care: an experimental study.** *BMJ Open* 2013.,**3**(4)**:** [http://bmjopen.bmj.com/content/3/4/e002700.full?rss=1] 10.1136/bmjopen-2013-002700 PMC3641486 23585394

[46] Cook R, Rasmussen J (2005). Going Solid: A model of system dynamics and consequences for patient safety. Qual Saf Health Care.

[47] Perry CAM, Hudson S, Ardis K (2011). “If I didn’t have anybody, what would I have done?: Experiences of older adults and their discharge home after lower limb orthopedic surgery. J Rehabil Med.

[48] Hesselink G, Link M, Olsson M, Parach P, dudzik-Urbaniak E, Orrego C, Toccafondi G, Kalkman C, Johnson KJ, Schoonhoven L, Vernooij- Dassen M (2012). Are patients discharged with care? A qualitative study of perceptions and experiences of patients, family members and care providers. BMJ Qual Saf.

[49] Popejoy L (2011). Participation of elder persons, families, and health care teams in hospital discharge destination decisions. Appl Nurs Res.

[50] Ocloo J, O’Shea A, Fulop N (2013). Empowerment or rhetoric? Investigating the role of NHS Foundation Trust governors in the governance of patient safety. Health Policy.

[51] Wiig S, Storm M, Aase K, Gjestsen MT, Solheim M, Harthug S, Robert G, Fulop N (2013). Investigating the use of patient involvement and patient experience in quality improvement in Norway: rhetoric or reality?. BMC Health Serv Res.

[52] Longtin Y, Sax H, Leape L, Sheridan ES, Donaldson L, Pitter D (2010). Patient Participation: Current Knowledge and Applicability to patient safety. Mayo Clin Proc.

[53] Plesk EP, Greenhalgh T (2001). The challenge of complexity in health care. BMJ.

[54] Kannampallil GT, Schauer FG, Cohen T, Patel LV (2011). Considering complexity in healthcare systems. J Biomed Inform.

[CR55] The pre-publication history for this paper can be accessed here:http://www.biomedcentral.com/1472-6963/14/365/prepub

