# Supplementary material for: Hospital discharge of the elderly-an observational case study of functions, variability and performance-shaping factors
Source: BMC Health Serv Res. 2014 Aug 30;14:365. doi: 10.1186/1472-6963-14-365 (PMC4159506; doi:10.1186/1472-6963-14-365)
Supplement: Supplementary file 1 — Additional file 1: STROBE Statement—checklist of items that should be included in reports of observational studies. (DOC 88 KB) [file 12913_2014_3462_MOESM1_ESM.doc]

STROBE Statement—checklist of items that should be included in reports of observational studies

|  | Item No | Recommendation |
| --- | --- | --- |
| **Title and abstract** | 1 | (*a*) Indicate the study’s design with a commonly used term in the title or the abstract  The study`s design: observational case study |
|  |  | (*b*) Provide in the abstract an informative and balanced summary of what was done and what was found  The abstract provides a short and balanced summary of methods and results |
| Introduction | | |
| Background/rationale | 2 | Explain the scientific background and rationale for the investigation being reported  We argue that mainstream patient safety research has tended to be reactive, investigating adverse events to identify cause- and-effect- relationships. Thus far little consideration has been afforded to investigate variability in everyday practices and how this can prospectively create system vulnerabilities. One of the study`s main objective is to explore and provide knowledge about performance variability in hospital discharge practices of the elderly. |
| Objectives | 3 | State specific objectives, including any prespecified hypotheses  The main aims of the paper are to identify;   - The functions of hospital discharge; - The areas of variations within those functions, and; - The performance shaping factors (PSFs) that may explain those variations. |
| Methods | | |
| Study design | 4 | Present key elements of study design early in the paper  Key elements of study design, methods applied, setting, sample inclusion and selection of participations are presented in the methods section. |
| Setting | 5 | Describe the setting, locations, and relevant dates, including periods of recruitment, exposure, follow-up, and data collection  Information on study setting and procedure for data collection are included in the methods section. |
| Participants | 6 | (*a*) *Cohort study*—Give the eligibility criteria, and the sources and methods of selection of participants. Describe methods of follow-up  (not applicable as the study is not a cohort study)  *Case-control study*—Give the eligibility criteria, and the sources and methods of case ascertainment and control selection. Give the rationale for the choice of cases and controls (not applicable as the study is not a case-control-study )  *Cross-sectional study*—Give the eligibility criteria, and the sources and methods of selection of participants (not applicable as the study is not a cross-sectional- study) |
|  |  | (*b*) *Cohort study*—For matched studies, give matching criteria and number of exposed and unexposed  (Not applicable )  *Case-control study*—For matched studies, give matching criteria and the number of controls per case  (Not applicable) |
| Variables | 7 | Clearly define all outcomes, exposures, predictors, potential confounders, and effect modifiers. Give diagnostic criteria, if applicable  (Not applicable) |
| Data sources/ measurement | 8* | For each variable of interest, give sources of data and details of methods of assessment (measurement). Describe comparability of assessment methods if there is more than one group  (Not applicable) |
| Bias | 9 | Describe any efforts to address potential sources of bias  Potential sources of bias are addressed under the section study limitations. |
| Study size | 10 | Explain how the study size was arrived at  Sample and selection of participants and settings are justified in the method section. |
| Quantitative variables | 11 | Explain how quantitative variables were handled in the analyses. If applicable, describe which groupings were chosen and why  Not applicable as the study does not include quantitative variables. |
| Statistical methods | 12 | (*a*) Describe all statistical methods, including those used to control for confounding  Not applicable |
|  |  | (*b*) Describe any methods used to examine subgroups and interactions  Not applicable |
|  |  | (*c*) Explain how missing data were addressed  Not applicable |
|  |  | (*d*) *Cohort study*—If applicable, explain how loss to follow-up was addressed  *Case-control study*—If applicable, explain how matching of cases and controls was addressed  *Cross-sectional study*—If applicable, describe analytical methods taking account of sampling strategy  Not applicable |
|  |  | (*e*) Describe any sensitivity analyses’  Not applicable |

Continued on next page

| Results | | |
| --- | --- | --- |
| Participants | 13* | (a) Report numbers of individuals at each stage of study—eg numbers potentially eligible, examined for eligibility, confirmed eligible, included in the study, completing follow-up, and analysed  Not applicable |
|  |  | (b) Give reasons for non-participation at each stage  Not applicable |
|  |  | (c) Consider use of a flow diagram  Not applicable |
| Descriptive data | 14* | (a) Give characteristics of study participants (eg demographic, clinical, social) and information on exposures and potential confounders  Not applicable |
|  |  | (b) Indicate number of participants with missing data for each variable of interest  Not applicable |
|  |  | (c) *Cohort study*—Summarise follow-up time (eg, average and total amount)  Not applicable |
| Outcome data | 15* | *Cohort study*—Report numbers of outcome events or summary measures over time  Not applicable |
|  |  | *Case-control study—*Report numbers in each exposure category, or summary measures of exposure  Not applicable |
|  |  | *Cross-sectional study—*Report numbers of outcome events or summary measures  Not applicable |
| Main results | 16 | (*a*) Give unadjusted estimates and, if applicable, confounder-adjusted estimates and their precision (eg, 95% confidence interval). Make clear which confounders were adjusted for and why they were included  Not applicable |
|  |  | (*b*) Report category boundaries when continuous variables were categorized  Not applicable |
|  |  | (*c*) If relevant, consider translating estimates of relative risk into absolute risk for a meaningful time period  Not applicable |
| Other analyses | 17 | Report other analyses done—eg analyses of subgroups and interactions, and sensitivity analyses  Description of the various phases of data analysis is presented in the method section. |
| Discussion | | |
| Key results | 18 | Summarise key results with reference to study objectives  Key results are summarised in the discussion section |
| Limitations | 19 | Discuss limitations of the study, taking into account sources of potential bias or imprecision. Discuss both direction and magnitude of any potential bias  Study limitations are addressed |
| Interpretation | 20 | Give a cautious overall interpretation of results considering objectives, limitations, multiplicity of analyses, results from similar studies, and other relevant evidence  Overall interpretation of results are given in the discussion section |
| Generalisability | 21 | Discuss the generalisability (external validity) of the study results  Issues concerning generalizability of study findings are raised under the section study limitations |
| Other information | | |
| Funding | 22 | Give the source of funding and the role of the funders for the present study and, if applicable, for the original study on which the present article is based  Information concerning funding is included in acknowledgements |

*Give information separately for cases and controls in case-control studies and, if applicable, for exposed and unexposed groups in cohort and cross-sectional studies.

**Note:** An Explanation and Elaboration article discusses each checklist item and gives methodological background and published examples of transparent reporting. The STROBE checklist is best used in conjunction with this article (freely available on the Web sites of PLoS Medicine at http://www.plosmedicine.org/, Annals of Internal Medicine at http://www.annals.org/, and Epidemiology at http://www.epidem.com/). Information on the STROBE Initiative is available at www.strobe-statement.org.
